# Left ventricular hipertrophy and ultrasound at emergency department

**DOI:** 10.1186/2036-7902-6-S1-A11

**Published:** 2014-01-31

**Authors:** A Oviedo-García, M Algaba-Montes, A Segura Grau, J Lopez-Libano, JM Alvarez-Franco, N Diaz-Rodriguez, A Rodriguez-Lorenzo

**Affiliations:** 1 Emergency Department, Valme Hospital, Seville, Members of the Working Group of Ultrasound SEMES_Andalucía and SEMERGEN, Spain; 2 MFYC, Médico ecografista en Centro Diagnostico Ecográfico y en Unidad de ecografía general del Hospital San Francisco de Asis, Madrid, Spain; 3 Critical Care Department, Miramar Hospital, Mallorca. Member of the Working Group of Ultrasound SEMERGEN, Spain; 4 Emergency Department, IB-Salut, Ibiza, Member of the Working Group of Ultrasound SEMERGEN, Spain; 5 Primary Care, Barbadás Primary Care Center, Ourense, Member of the Working Group of Ultrasound SEMERGEN, Spain; 6 Radiology Department, Perpetuo Socorro Hospital, Vigo, Member of the Working Group of Ultrasound SEMERGEN, Spain

## Background

in Spain, the skills of Emergency Physicians (EP) in the process of echocardiography have been discussed for more than three decades. The current scientific evidence supports strongly the use of echocardiography by the EP, for its speed, agility and safety for the patient providing an early diagnosis of serious or potentially serious diseases. In this sense, echocardiographic technique training makes that in the clinical practice of Emergency Departments is used as diagnostic support tool in initial attention to the patient suffering.

## Objective

To train in the management and diagnosis of the echocardiographic technique among professionals in the ED, and promoting their use on the basis of the advantages that this presents, due to its characteristics of safety, efficiency and safety for the patient.

## Patients and methods

The diagnosis of the left ventricle hypertrophy by echocardiographic evaluation. We used and ultrasound Sonosite M-Turbo, equipped with P21 between 1 and 5 MHz probe.

## Results

When a study shows left ventricle hypertrophy, we must stop to assess two important details:

2. The diastolic function: In the hypertrophic ventricles, in which performance is decreased, wave E has a slower curve for deceleration and A wave is higher, which means a worse diastolic funtion.

3. The presence of dynamic obstruction of the outflow tract, which can go with previous systolic motion of the previous mitral valve.

## Conclusion

To incorporate echocardiography at ED decreases overall care times, since the EP are more effective, efficient and dynamic in the management of emergency "time-dependent", providing a greater clinical safety.
